# Self‐care neglect through the voices of nurses working in primary healthcare clinics in Gauteng, South Africa

**DOI:** 10.1111/nuf.12812

**Published:** 2022-10-13

**Authors:** George Nkabinde‐Thamae, Charlene Downing, Sanele Nene

**Affiliations:** ^1^ Department of Nursing University of Johannesburg Johannesburg South Africa

**Keywords:** mindfulness, self‐care, well‐being

## Abstract

**Background:**

Self‐care is essential, but while professional nurses often pay attention to the health of their patients, they give little heed to their own well‐being. With the current pandemic continuing to negatively affect the world, the need for health professionals to make time for self‐care is imperative. The concept “self‐care” is not a new phenomenon; however, this study strives to show the importance of self‐care practices in the world of nurses and its benefit for the nursing profession. The reality for nurses taking care of themselves will assist them in providing consistent quality care for their patients.

**Method:**

A qualitative approach with a descriptive, phenomenological, contextual method was used in this study. Ten professional nurses employed within different primary healthcare clinics were selected through purposeful sampling. Through in‐depth, individual interviews, the professional nurses shared their lived experiences with self‐care practices while being employed within a primary healthcare clinic. The recorded interviews were transcribed verbatim and then analyzed using Colaizzi's method.

**Results:**

The findings revealed:

- Theme 1: Participants experienced internal and external factors that compromised self‐care practices
- Theme 2: Holistic well‐being and the quality of patient care are compromised by self‐care neglect
- Theme 3: Participants experienced the need to take responsibility and accountability to promote self‐care practices.

**Recommendations:**

Specific recommendations were formulated to facilitate professional nurses' empowerment to practise self‐care as a lifestyle. These specific recommendations focused on reducing the burden of caring for others to the extent that professional nurses working in primary healthcare settings have nothing left for themselves.

## 1 INTRODUCTION

The researchers became aware that self‐care practices were fragmented in professional nurses' lives and were exacerbated by the Covid‐19 pandemic. The unique contribution of this study is that it focuses on nurses' well‐being and empowerment to practise self‐care, so they experience better health. Lubinska‐Welch et al.^1^ claim self‐care practices entail an individual consciously practising healthy behaviors to achieve optimal health. This study was conducted in the context of nursing, a caring profession practised by a person registered under section 31 of the Nursing Act 33 of 2005. These individuals support, care for and treat healthcare users to achieve or maintain optimal health South African Nursing Council.^2^ Crane and Ward^3^ state that even though many view nursing as a caring profession, is it also evident that professional nurses neglect their own well‐being. Literature provided by Downing and Hastings‐Tolsma^4^ and Jooste^5^ similarly states that caring within the nursing context links to the philosophy of Ubuntu, expressed as human kindness to others and patient fulfillment. Further, Watson^6^ emphasized that caring is regarded as a shared experience in which professional nurses and patients interrelate to ensure the overall and total health of the patient. In support, Collins^7^ reveals caring is a feeling and expression of concern and empathy for others, and Jooste^5^ alludes that caring is practised by demonstrating kindness and concern for others, distinguished by love, support, and the environment. The mentioned literature illustrates that it appears as if nurses, in general, might feel they should be caring for others and not for themselves, potentially promoting nurses' fragmented self‐care practices.

The first researcher observed that nurses often care for patients while compromising their own well‐being. During the Covid‐19 pandemic, nurses worked particularly long hours, and skipped meals and lunch breaks due to the demands the healthcare setting faced. Huang et al.^8^ found that despite the pandemic, healthcare workers remained in clinical practice, yet they felt stressed and demonstrated anxiety as they were afraid of contracting the virus and infecting their families. Kang et al.^9^ also narrated that nurses felt it was their role and obligation to care for patients, even in dangerous situations. Moreover, nurses are vulnerable to compassion fatigue because of the nature of their work, long working hours, inadequate equipment, and poor working conditions, which harm their health.^10^ Dempsey and Reilly^11^ also draw attention to self‐care neglect among professional nurses, and claim it appears to be related to emotional exhaustion, compassion fatigue, and uncaring practices toward themselves. Professional nurses seem to find it difficult to promote and uphold their well‐being, though they pay attention to the health of their patients.^12^, ^13^ Leão et al.^14^ also agree that self‐care practices appear to be neglected by most healthcare professionals as they primarily focus on others' well‐being.

## 2 METHOD

To recruit information‐rich participants, researcher one provided the healthcare facilities with posters and flyers containing information about the study and his contact details, approximately 23 professional nurses from different healthcare facilities, showed interest in the study, but only 10, consented and participated. The professional nurses who decided not to partake in this study were not punished in any way. Further unstructured individual interviews were considered an appropriate data collection method as it assisted the researchers in exploring professional nurses' experiences with self‐care practices. The authors found Husserl's approach appropriate for this study and bracketed their own natural attitudes. Ten participants were selected to share their individual experiences with self‐care practices.^15^ Further, the researchers applied Heidegger's philosophical viewpoint, which empowered and unveiled greater insight into professional nurses' lived experiences with self‐care practices.^16^, ^17^

### 2.1 Objective

This study was conducted using a descriptive qualitative approach

- to explore and describe professional nurses' lived experiences of practising self‐care; and
- provide recommendations to facilitate professional nurses' empowerment to practise self‐care as a lifestyle.

### 2.2 Study design

This qualitative study was conducted using Colaizzi's phenomenological approach to determine professional nurses' lived experiences with self‐care practices while employed within a primary healthcare (PHC) clinic. Colaizzi's phenomenological approach focused on the individuals' emotions and experiences rather than measuring the phenomenon.

### 2.3 Participants and data collection

Sampling was performed using the purposive sampling method. To gather data, unstructured individual interviews were conducted through a conversation with a purpose.^18^ The research environment was PHC facilities in Gauteng, and the place of the interviews was determined in coordination with the participants, with strict adherence to Covid‐19 regulations. One pilot interview was conducted. Each interview was approximately 45–60 min and lasted until sufficient data were gathered. The first researcher conducted all 10 interviews to increase the data's integrity. Moreover, the number of participants was not predetermined; sampling continued until data saturation was reached, which was achieved by the 10th interview.^19^

One central question was posed to all participants to eliminate the researchers' bias and presumptions. Further probing questions were asked; for example, “What are your experiences with self‐care practices?” To engage with the participants, different open‐ended questions were employed to understand their lived experiences; for example, “how does not eating healthy relate to self‐care neglect?” “What consequences will there be if you practise self‐care?” The information was elicited from the participants in a respectful and sensitive manner. All questions had to be clear to the participants so the researchers could fully understand the participants' world and relationship with self‐care practices.

With self‐care practices not being a new phenomenon, the researchers also had to ensure the research question fitting for this study was not asked in a similar or different context. To exclude this possibility, the researchers searched other published articles using Google Search, Google Scholar, and the University of Johannesburg database. Different keywords were used, including “self‐care,” “diet and wellness,” “nurses,” and “compassion fatigue.” Recent literature on self‐care by Hossain and Clatty,^20^ Chipu and Downing,^21^ and Brouwer et al.^22^ were read in depth, and it was determined that despite self‐care practices being compromised in the world of nurses, no evidence reflected that a similar question had previously been asked.

### 2.4 Data analysis

Colaizzi's seven‐stage data analysis process, as presented by Morrow et al.,^23^ was followed as stipulated below:

- In the first stage, each interview was recorded, transcribed verbatim, and reviewed several times to understand the whole.
- In the second stage, meaningful sentences were extracted by the underlining method.
- In the third stage, initial codes were extracted.
- In the fourth and fifth stages, the developed concepts were divided into subconcepts, and the results were turned into main themes, respectively.
- In the sixth and seventh stages, a comprehensive and unambiguous description of the phenomenon was developed, and then validated by presenting some of the results and extracted concepts to the participants (member check) to be confirmed by them. In case of ambiguity, some corrections were made. The first author and an independent coder did the coding simultaneously and independently.

### 2.5 Trustworthiness

Considerations of rigor and trustworthiness were addressed by adopting Guba and Lincoln's^24^ guidelines. Credibility was established using member and peer‐checking, reviewers' prolonged engagement with data, and maximum variance in participant selection. Regarding member‐checking, part of the interviews and codes were also presented back to the participants for review to ensure that the data reflect their real experiences and perceptions. The researchers considered transferability by providing a detailed description of data collection, analysis processes, and findings. This method enhances the applicability and generalizability of results in similar contexts.

## 3 RESULTS

The results derived from this study were obtained from professional nurses employed within different primary care clinics. The context in which the study was conducted has approximately 33 healthcare facilities. However, in this study, 10 professional nurses from different healthcare facilities participated, five participants were female, and five were male. The participants' ages ranged from 30 to 51 years, and they had vast working experience, from 4 to 25 years in PHC clinics. Participants either had a degree or diploma in nursing (general nursing science, community, psychiatry, midwifery). The findings revealed that professional nurses struggled with self‐care practices. Internal and external factors seemed to be contributing toward them neglecting themselves. It was also evident that professional nurses unconsciously neglected themselves and prioritized caring for their patients, family, and community. Moreover, professional nurses' self‐care neglect seemed to indirectly affect patients' quality of care. The professional nurses' health was on the verge of crisis, and time for their loved ones and themselves was nonexisting.

These findings painted a picture of nursing as an unbearable and unsafe occupation. Professional nurses who took part in this study shared they did not know whether to stay or leave the nursing profession. Three themes emerged after data analysis. Theme 1: Participants experienced internal and external factors that compromised self‐care practices. Theme 2: Holistic well‐being and the quality of patient care are compromised by self‐care neglect. Theme 3: Participants experienced the need to take responsibility and accountability to promote self‐care practices. As researcher one collected the data, he received great insight into participants' lives and had a closer ear to hear, sense, and understand their lived experience of self‐care practices. It was evident that self‐care was on these participants' wish lists, but it appeared practically impossible in their lives.

### 3.1 Theme 1: Participants experienced internal and external factors that compromised self‐care practices

The participants shared that they lived in a reality where various internal and external factors hindered them from practising self‐care. During the interviews, participants explained that their daily circumstances compelled them to put the needs of others first, neglecting their own. The internal and external factors mentioned by the participants included subconscious self‐negligence, ignorance, and an inability to make time for self‐care.

#### 3.1.1 Category 3.1.1: The participants subconsciously neglected themselves: Who comes first—A nurse or a patient?

The participants expressed they did not practice self‐care; they unknowingly decided to make their patients a priority, as a result neglecting themselves. In all the interviews, it became clear that patients and others were always prioritized, and nurses were constantly neglecting themselves. It appeared as if nurses had to be given permission to practise self‐care, because they feared if they did, there would be unknown consequences to them.

The following narrative was shared:

The truth is if I decide to practice self‐care there will be consequences, so I constantly choose to self‐neglect, and be there for the patient, and everybody else. They say nurses are Angles on earth, so I am a male angel on earth, a very tired one I must say. (Participant 10, Male, 32)

During the data collection process, researcher one became mindful that self‐care practice was not a new concept to the participants, but it sounded as if they were practising self‐care in a fragmented manner. A participant stated:

I believe that I do practice, self‐care aspects, but not holistically. It's not healthy, it's not normal actually, but because one has become so selfless, that one normally doesn't think about themselves but the next person. Even at home you end up thinking, putting other people first, whether friendships, whether relationships. (Participant 2, Male, 34)

Another participant expressed her hindrance with self‐care practices:

Ignorance and time has contributed towards me not being able to practice good care towards myself. (Participant 5, Female, 51)

Professional nurses' lived experiences while employed within a PHC setting reflected that self‐care seemed impossible due to the demands of the nursing profession. The input from these two participants spoke volumes:

So its demanding where it comes to a point where you really neglect your own needs, its demanding to a point even if you are sick, you sick but you still say if I don't go to work, already we a limited number of professional nurses at work, if I don't go there will be more strain on my colleagues. (Participant 1, Female, 34)

I am neglecting myself, I am not taking care of myself, it starts affecting most areas of your life, whilst even if people take advantage of you, you would not even notice that the next person is takingS advantage of me, because it has become a norm, and it is normal. (Participant 2, Male, 34)

These narratives illustrate that the participants struggled to practice self‐care in their daily lives. Despite participants sharing their own, real‐life stories, their narratives were similar, and it became evident that they neglected themselves, not seeing the need or the importance of practising self‐care, as they felt responsible and accountable for taking care of those around them. The participants struggled internally to make a conscious decision to practice self‐care; they viewed it as being selfish since the demands of their profession required them to practice compassion for others. However, in the end, it sounded as if they experienced compassion fatigue.

### 3.2 Theme 2: Holistic well‐being and the quality of patient care are compromised by self‐care neglect

The participants reported they experienced self‐care neglect, which compromised holistic well‐being. The participants themselves were suffering from chronic conditions but were so immersed in their daily duties that they had no desire to take ownership of their health. Instead, they continued to provide care for their patients and neglected their own well‐being.

#### 3.2.1 Category 3.2.1: Participants neglected their general health despite suffering from chronic diseases and unhealthy behaviors, including unhealthy dietary practices, which led to weight gain and obesity

Participants expressed that they struggled to take ownership of their own health. These participants suffered from chronic diseases, including hypertension and diabetes, but did not prioritize their health needs. The participants shared that they engaged in poor dietary practices, which further compromised their health; such behaviors eventually increased their weight and led to obesity. The following quotes emphasize this theme:

I am a person that has hypertension, and you will find at that given time I have not had my medication, but because I am more concerned about the patient, I do not really concern about that, then if I find myself I have headaches, I know the symptoms of hypertension. (Participant 4, Male, 40)

I am on chronic medication, and because we are always busy at work my patients need me…, at times I even forget to take my medication, as prescribed by the Doctor (Participant 3, Female, 45)

Despite the professional nurses being knowledgeable of the dangers and complications of uncontrolled chronic diseases, they still did not prioritize their health, but saw self‐medication as an alternative. The danger of such practices was that they took medication without proper examinations. This reality was revealed by the direct quote:

If not often prioritize not going for my annual check‐ups or even routine checkups. But I guess…san example this past weeks the weather is not ok, my chest gets tight and breathing becomes difficult, but I am also PHC trained so I self‐prescribe medication. (Participant 6, Male, 39)

These verbatim quotes are shocking but were a true interpretation of these three participants' experiences. The link between diet and self‐care neglect was also elaborated on in by the following participant:

Remember I overweight, I just get to work I just push the queues, so eventually you start eating when everybody is gone, then you just have something lite to snack on. I stay by myself…I am too tired to be standing in front of a stove and cook, no … so I rather stop at Chicken Licken, so what does the Chicken Licken do, will lead to more obesity, you don't even have strength to go to the gym. You rather buy Mac Donald's, just sit in front of the TV, and little by little you gain more weight … this leads to physical impairment. Aches and pains, this leads to other chronic diseases, hypertension and diabetes, heart or cardiac conditions, this is all contributed to self‐care neglect. In the end of the day I am still not self‐caring, because a nurse that practice self‐care, would have a healthy breakfast, have her tea, have at least 8 glasses of water per day, but I would rather buy 2 liters of coke, what we do we find comfort with food, and when you are tired, you don't have the strength to cook, food will be the take outs … nice take outs, leads to weight gain. (Participant 8, Female, 37)

Poor dietary intake and its consequences were elaborated on by participant eight; despite the participant having information on the impact of a poor diet on her health, she continued to engage in such unhealthy practices. Ultimately, self‐care is imperative in the nursing profession, and the following quote offers additional insight into the extent to which nurses neglect self‐care practices:

I think i neglected me, i neglected caring for me, i neglected loving myself, i neglected many things about me. (participant 5, female, 51)

Listening closely to what the participants shared, it sounded as if they were mindful that self‐care neglect did not have a positive impact on them. Fragmented self‐care practices contributed to nurses being physically, mentally, and spiritually tired, affecting the quality of care they provided their patients. This phenomenon illustrated that self‐care practices would be beneficial to both the patients and nurses.

The following narratives emphasize that self‐care neglect among professional nurses affects patient care:

Definitely, to be honest no … yes you give them the medication, but to actually having an actual conversation with the patient … there is no time to ask a patient how they are doing, or to interact with them, a patient might come for their diabetic medication, but in the meantime they are suicidal, and you don't even pick up on that, because of the rush, rush. We do not nurse a patient holistically, there is just no time, and at college, they taught us to nurse a patient holistically. (Participant 8, Female, 37)

You see in a nutshell self‐care is not possible because at work is the patient and the day‐to‐day running of the facility, you see even if you are putting the patient first you are still not even providing quality of care, because we doing everything in rush, half done … because it's not like we at work the entire day, so even if you put the patient first, but those very same patients don't get the best of our care … Quality of care … comprehensive care, that's why despite I feel I give a lot of me to my patient, there is still a lot complaints that's due to self‐care neglect. (Participant 7, Female, 49)

The patients under my care indirectly are unsafe because they are nursed by someone that's there in the flesh, but death or absent mentally, so mistakes in terms of giving out wrong medication can happen, and a patient can actually die, due to negligence. You see there is many things that prevents one to practice or try to practice self‐care. (Participant 9, Male, 30)

This theme reflects the undesired consequences of self‐care neglect on the individual professional nurse, their relationships with themselves, and the quality of care the patients receive due to professional nurses not practising self‐care in totality. The researchers also concluded from these quotes that nurses practising fragmented self‐care might contribute to possible litigations within the healthcare sector.

### 3.3 Theme 3: Participants experienced the need to take responsibility and accountability to promote self‐care practices

The participants realized they have to take ownership of their lives to practice daily self‐care. The interviews reflected how determined the participants were to start practising self‐care. Participants shared what intervention strategies they planned to attempt to practice self‐care.

#### 3.3.1 Category 3.3.1: Participants were aware of programmes at work but expressed a deeper yearning for alternative practices of self‐care

During the interviews, participants explained there are employee wellness strategies at their work. However, despite the availability of these wellness programmes, they had the urge to explore alternative strategies. The participants came to understand the importance of self‐care practices and interventions needed to assist them.

The following quotes reflect participants' realization of the need for self‐care strategies:

- “I would also be more gentle with myself, forgive myself and set boundaries where my family is concerned I should be able to say No, and make time to spend time with me, even if it's reading a book, spending some time in the garden.” (Participant 7, Female, 49)
- “Allowing nurses to also reflect on their psychological well‐being, one could have been in a situation whereby you could debrief whilst at work, by asking advice and clarity from your colleagues.” (Participant 9, Male, 30)
- “I feel overwhelmed so writing things out, will help me to let everything out that might be heavy on my soul, spirit and as result causing me to self‐neglect, so having a journal…will assist in debriefing…. Yes, debriefing.” (Participant 6, Male, 36)

These narratives unveiled the participants' mindfulness in taking ownership, responsibility and accountability for practising self‐care. A vivid snapshot was presented of the directions these individual professionals were willing to embark on to practice self‐care in their lives. They could see, feel and sense the negative consequences of not practising self‐care, and they were eager to put in the work, starting with themselves.

## 4 DISCUSSION

In the lives of these participants, self‐care practices appear to be impossible. Instead, their focus is on caring and making others a priority. Perez^12^ agreed that self‐care is not always possible among professional nurses because these individuals are used to taking care of others first and putting themselves last. Crane and Ward^3^ also claimed that most professional nurses feel it is selfish to practise self‐care. According to Austen,^25^ nurses put their self‐care priorities last, even if it is just basic activities that may revive or reinstate their general well‐being. Padilha et al.^26^ further allude that caring for other people's needs has always been a priority in nursing theories, as nurses typically care for people from birth until death.

The researchers could sense that self‐care neglect among these participants was an unconscious decision, as it appeared they prioritized their patients out of love for them. However, while professional nurses' main priority is their patients and helping others, it predisposes them to compassion fatigue and decreases their quality of life.^27^, ^28^ Ultimately, the reality of self‐neglect among nurses appears to have a negative impact on the individual nurse and the patients attending the PHC clinic.

The participants shared many aspects of their lives where they experienced gaps in practising self‐care. However, according to Ramos,^29^ nurses mainly neglect self‐care practices such as healthy eating habits or their general well‐being due to making the demands of the profession and the needs of patients their only priority. Nurses seem to struggle to allocate or make time to care for themselves or engage in activities that may sustain or promote their health.^30^ Hilli and Eriksson^31^ emphasize that nurses need to focus on making their own health a daily priority to benefit them and their patients. A study by Steinwedel^32^ determined that it should be a professional and moral duty for nurses to care for their physical, spiritual, and psychosocial well‐being.

In this study, the researchers were in a position to understand that nurses loved their occupation, but self‐care neglect contributed to their desire to leave the nursing profession. This view is also supported by Arimon‐Pagés et al.,^33^ who revealed an increase in nurses' absenteeism, and the desire to quit their current position or the profession entirely becomes a constant feeling of anxiety among a majority of nurses, due to their working conditions. The lack of self‐care practices caused participants' lives to spiral out of control, to the extent that it affected their eating habits and general health.

Kelly and Wills^34^ and Kyle et al.^35^ substantiate that a lack of self‐care among nurses has contributed to nurses suffering from high levels of obesity, which makes them more prone to chronic diseases, such as hypertension and diabetes. According to Torquati et al.,^36^ nurses adhering to poor nutritional intake are at an increased risk for chronic health problems. Similarly, Sala‐Vila et al.,^37^ found that adequate fruit and vegetable consumption is beneficial to individual nurses, though few engage in this practice. Consuming adequate fruits and vegetables promotes healthy eating habits and decreases nurses' mortality rate. However, Younas^13^ alludes that despite nurses having in‐depth knowledge of health‐promoting activities, they still struggle to indulge in healthy activities, such as regularly exercising, refraining from smoking, and practising healthy eating habits. Ultimately, Heidari et al.^38^ and Ross et al.^39^ conclude that such inherent practices will further lead to obesity, musculoskeletal problems, inactive lifestyles and burnout. Studies conducted by the World Health Organization^40^ provided evidence that over 63% of deaths among nurses are related to uncontrolled diabetes, hypertension, and ischemic heart disease, all of which could be prevented if nurses engaged in regular physical activity and healthy eating habits. Kelly and Wills^34^ also shared that obesity among nurses could result in workplace injury, loss of productivity, sickness, high rates of absenteeism, and affect the mental well‐being of the individual nurse. During data collection and analysis, it became clear that a majority of the participants were on chronic medication; however, they did not take their own health seriously, but instead took self‐prescribed medication without any medical examination, which appeared not to improve their health.

Effective treatment and routine medical check‐ups are imperative to promote more effective and longer‐term prevention of cardiovascular disease, resulting in greater protection of the workforce capacity.^41^, ^42^ Khalil and Hamdan‐Mansour^43^ further assert that nonadherence to an individual's treatment plan, including medication and other health activities, will compromise their well‐being and social, physical and psychological functioning. A study by Khatony et al.^44^ reported that nurses and nursing students often decide to self‐medicate because they want to avoid consulting doctors. They seldom take their illnesses seriously and do not have time for consultations. Moreover, some attempt to diagnose and treat themselves as they think they have sufficient pharmacological knowledge. According to Linton and Koonmen,^45^ nurses' inability to care for themselves will have a negative impact on their general health and the quality‐of‐care patients receive. In addition, Sagherian et al.^46^ reported that nurses who do not practice self‐care are more prone to experiencing acute or chronic fatigue. The danger is that these nurses will be less alert and suffer decreased concentration levels, potentially making mistakes and compromising patient care.

Grøndahl et al.^47^ disclosed that the quality of healthcare patients receive within the healthcare sector is compromised and unsatisfactory, as expressed by a majority of patients in the private and public sector. Patients are unhappy and concerned about healthcare services due to long queues, unavailability of medicines, shortage of nurses, and nurses' uncaring attitudes, expressed in the form of shouting and improper communication with patients.^48^ Anderson et al.^49^ and Hastings‐Tolsma et al.^50^ also provided evidence that patients in South Africa experience verbal and physical abuse from nurses because of heavy workloads, limited engagement with patients, and inadequate or limited staffing. These challenges ultimately impact the total health of the patients and nurses, resulting in self‐care neglect and poor health. Literature illustrated that patients were not receiving quality patient care because nurses were running on empty. The participants also came to the realization just how self‐care neglect has affected their daily living, and they were eager to change their lack of self‐care practices.

A study conducted by Anheyer et al.^51^ revealed some useful strategies nurses and nursing students could use in practising self‐care. These include using a reflective journal to assist them in identifying problems and being able to solve them, which will promote calmness and decrease stress in their daily lives. Andrews et al.^52^ assert that strategies to assist nurses in promoting their own well‐being will only be successful if nurses are able to be self‐caring and self‐compassionate. Nurses are aware that they need to take care of themselves but struggle to incorporate self‐care practices in their day‐to‐day lives; nurses must dedicate time for their own well‐being.^53^ These authors noted that many nurses have more than one job that contributes to their monthly income, which prevents them from having time for themselves. This may be perceived as a lack of priority toward themselves, as they constantly experience anxiety, agitation, and exhaustion. Caring for the self will help individuals engage positively with those around them and their environment.^53^

## 5 LIMITATIONS

This study was limited to professional nurses employed within PHC clinics in Gauteng Province. Other categories of healthcare workers, such as medical doctors, enrolled nurses, and auxiliary nurses were excluded, limiting the overview of healthcare workers' lived experiences with self‐care practices. The Covid‐19 pandemic may also have significantly impacted the overall findings and self‐care practices.

## 6 CONCLUSION

This exploratory qualitative study gave professional nurses an opportunity to voice their experiences of self‐care practices while working at a PHC clinic. When participants shared their self‐care practice stories, which appeared as a gap in their real‐life world, the researchers' interactions with these participants emphasized the need for individuals to take accountability and responsibility for practising self‐care.

### ETHICS STATEMENT

Before proceeding with this study, the researchers obtained approval from various independent ethics committees.^54^ The University of Johannesburg, Faculty of Health Science, the Research Ethics Committee, NHREC (Registration number: REC‐241112‐035, REC Clearance number: REC‐504‐2020) and the Higher Degrees Committee (HDC −01‐22‐2020) approved the study; certification was issued to the researchers. To enter the field to collect data, permission was also received without objection from the Ekurhuleni Health District Research Ethics Committee (NHRD No: GP‐20200‐032). The researchers obtained informed consent from the participants before entering them into the study, gave participants the right to withdraw from the study at any time, maintained anonymity and confidentiality of information, and provided results to the participants at their request.

## Supporting information

Supporting information.

## Data Availability

The data that support the findings of this study are available from the corresponding author upon reasonable request.
