# Supplementary material for: Self‐care neglect through the voices of nurses working in primary healthcare clinics in Gauteng, South Africa
Source: Nurs Forum. 2022 Oct 13;57(6):1330–8. doi: 10.1111/nuf.12812 (PMC10092092; doi:10.1111/nuf.12812)
Supplement: Supplementary file 1 — Supporting information. [file NUF-57-1330-s001.docx]

**Research Ethics: Clearance**

Before proceeding with this study, the researchers obtained approval from various independent ethics committees (Creswell & Poth, 2018:54). The University of Johannesburg, Faculty of Health Science, and the Research Ethics Committee, NHREC (Registration number: REC-241112-035) and the Higher Degrees Committee (HDC -01-22-2020) approved the study; certification was issued to the researchers. To enter the field to collect data, permission was also received without objection from the Ekurhuleni Health District Research Ethics Committee (NHRD No: GP-20200-032).

**Acknowledgements**

The researcher thanks all the participants for sharing their most personal and valued experiences.

**Author Contributions**

Study design: GNT, CD and SN

Data collection: GNT

Data analysis: GNT, CD and SN

Manuscript writing and revisions for important intellectual content: GNT, CD and SN
